# Quality of Life in Post-Surgical Hypoparathyroidism (PoSH) in Thyroid and Parathyroid Surgery

**DOI:** 10.1007/s00268-022-06730-7

**Published:** 2022-10-02

**Authors:** Sarah L. Hillary, Je Ern Chooi, Jonathan Wadsley, John D. Newell-Price, Nicola J. Brown, Saba P. Balasubramanian

**Affiliations:** 1 Department of Oncology and Metabolism, University of Sheffield, Sheffield, UK; 2 Sheffield Teaching Hospitals NHS Foundation Trust, Sheffield, UK; 3 Weston Park Hospital, Sheffield, UK

## Abstract

**Background:**

Post-surgical hypoparathyroidism (PoSH) is often long term, with significant associated morbidity and ongoing treatment. A recent systematic review found impaired quality of life (QoL) in patients with PoSH, despite stable treatment. Most studies did not include an appropriate control arm and further studies were recommended, taking into account underlying disease and comorbidities. This study aims to compare QoL in patients with PoSH with appropriate control groups.

**Methods:**

This was a cross-sectional observational study using the general quality of life SF-36 tool and a hypocalcaemia symptom score (HcSS) to assess QoL in patients with PoSH and controls (who had similar surgery but without PoSH). Participants were identified from two patient groups (the Butterfly Thyroid Cancer Trust and the Association for Multiple Endocrine Neoplasia Disorders) and a single tertiary centre in the UK.

**Results:**

Four hundred and thirty-nine responses (female *n* = 379, PoSH *n* = 89) were included with a median (range) age of 52 (19–92) years. Reported dates of surgery ranged from 1973 to 2019. HcSS scores showed significantly more associated symptoms in patients with PoSH than those without (*p* < 0.001). Although there was no overall difference in QoL between groups, patients with PoSH consistently had lower scores (*p* = 0.008) in the energy/fatigue subdomain of the SF-36.

**Conclusion:**

Patients with PoSH reported significantly more fatigue and loss of energy compared to appropriately matched controls, but overall QoL was not significantly different. Standardised QoL measures may not be sensitive enough to highlight the impact on QoL in these patients. A disease-specific tool may be required.

## Introduction

Intraoperative bruising, devascularisation or inadvertent excision of the parathyroid glands during neck surgery can result in low levels of parathyroid hormone (PTH) causing hypoparathyroidism and hypocalcaemia [1]. In most, hypocalcaemia is transient. Long-term (> 6 months) hypoparathyroidism is less common but associated with significant morbidity and potentially increased mortality [2–4].

Studies report that between 0 and 17.4% develop long-term hypoparathyroidism and the incidence of temporary hypocalcaemia varies between 0 and 46% [5, 6]. The variation in reported figures is attributed to different definitions of hypocalcaemia and hypoparathyroidism [6], differences in method of assessment and heterogeneity in extent of surgery. The fifth national audit by the British Association of Endocrine and Thyroid Surgeons (BAETS) reported a 23.6% incidence of transient hypocalcaemia and a 6.5% incidence of long-term hypocalcaemia after bilateral thyroid surgery [7].

Long-term hypoparathyroidism requires continuing treatment and is associated with renal stones, nephrocalcinosis and soft tissue calcification [8, 9]. In addition, there appears to be a significant impact on quality of life (QoL) [10]. A systematic review [11] found that patients with hypoparathyroidism (HPT) receiving standard treatment had impaired QoL compared to both the general population [12–14] and other matched patient controls [11, 15, 16]. It appears that patients, despite stable treatment with calcium and vitamin D supplements, still experience similar or lower QoL than other patients with long-term medical conditions [11, 15, 17, 18]. The studies included in this review [11] used generic questionnaires and may have overlooked some significant issues important to patients suffering with HPT. Only two studies in the review compared to matched cohorts [15, 16] in small numbers of patients (*n*  = 50 and *n*  = 44, respectively). The other studies used national reference data for comparison. Previous studies have concluded that the difference in QoL between patients with PoSH and the general population are due to PoSH. The aim of this study is to perform a detailed assessment of symptoms and QoL in patients with PoSH and to compare these patients with similar cohorts of patients without hypoparathyroidism, taking into account the aetiology of hypoparathyroidism.

## Methods

This is a cross-sectional observational ‘web-based’ questionnaire study of patients with PoSH and appropriate controls. Members of two charities, Butterfly Thyroid Cancer Trust (BTCT) and the Association for Multiple Endocrine Neoplasia Disorders (AMEND), were invited to participate. Also patients who underwent surgery for thyroid disease or for primary hyperparathyroidism at Sheffield Teaching Hospitals NHS Foundation Trust (STHNFT) from 2010 to 2016 were screened. Patients were included if they had a bilateral neck exploration or total thyroidectomy (*n*  = 875). Electronic records were reviewed to identify patients who needed treatment for over 6 months after surgery (*n*  = 16). Each of these patients were matched from the same database with three other patients of similar age (date of birth within 12 months of the index patient), sex and procedure in the index year (*n*  = 48). A total of 64 patients were invited to participate. Patients were then identified from thyroid cancer and MEN clinic schedules between 1 January 2017 and 31 December 2017 (*n*  = 534 and *n*  = 100, respectively). After exclusion of duplicates, deceased patients and those for whom clinical details including surgical records were not available, further 303 patients were identified.

Potential participants were invited by post to complete the online questionnaire through REDCap. Patients had the option to request a paper copy of the questionnaire. A participant information sheet and consent form were presented online.

The questionnaire included a section on demographic details: details of treatment, nature and duration of hypocalcaemia; and current medications: the SF-36 and the HcSS (hypocalcaemia symptom score [19]). The SF-36 is a 36-question short form health survey developed by RAND Healthcare, which is a verified, generic set of QoL measures [20]. The HcSS is an as yet unvalidated tool that includes symptoms directly linked with hypocalcaemia and associated comorbidity; this has been developed based on expert opinion and key members of the Parathyroid UK charity. The focus of this study was to evaluate the existing SF-36 and to explore the severity of symptoms using the HcSS and correlate this with QoL scores.

Patients were divided into PoSH and non-PoSH groups based on the BAETS definition of long-term hypoparathyroidism—requirement for treatment at six months after surgery [7]. With regard to supplements, alfacalcidol or calcitriol (active vitamin D) and not inactive vitamin D or calcium supplements were required to establish a diagnosis of PoSH.

The data were downloaded from the REDCap database onto a spreadsheet for descriptive analyses. Categorical data were described using frequencies (or percentages), and continuous data that were not normally distributed were described using median and interquartile range. Statistical analysis was performed using SPSS (IBM Corp. Released 2017. IBM SPSS Statistics for Windows, version 25.0. Armonk, NY: IBM Corp.). Differences in the SF-36 scores and HcSS between PoSH and non-PoSH groups were analysed with nonparametric tests including Mann–Whitney U test and Kruskal–Wallis’ test to compare the three pathological groups—thyroid cancer, MEN disease and benign pathologies. Spearman’s rho was used to evaluate the correlation between the SF-36 subdomains and HcSS scores. Differences were considered statistically significant at *p* < 0.05. The project was approved by the Research Ethics Committee (REC Reference: 18/ES/0091, IRAS Project ID: 245,001) and registered with the R&D department in Sheffield Teaching Hospitals (STH reference: STH 20,208). Informed consent was obtained prior to access to the questionnaire.

## Results

**Fig. 1:**
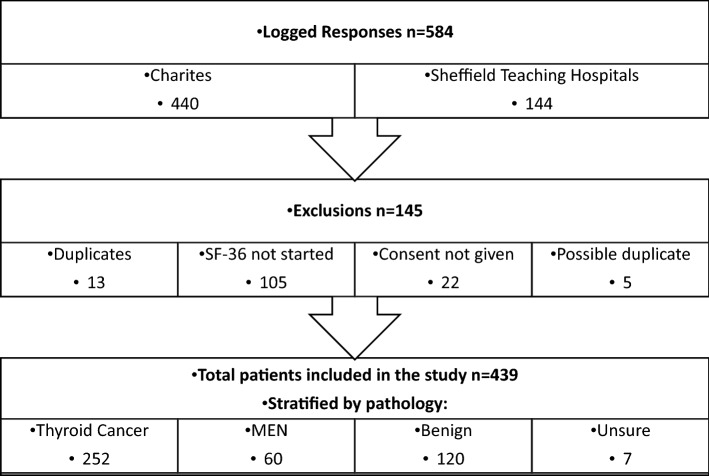
Flow chart demonstrating recruitment of the different cohorts and classification into different groups by pathology

The REDCap website logged 584 responses from four sources (Fig. 1) [21]. Of these, 145 responses were excluded (potential duplicate responses (*n* = 18), SF-36 not completed (*n*  = 105) and consent not given (*n*  = 22)). The remaining 439 responses were stratified by pathology—thyroidectomy for cancer (*n*  = 252), MEN (*n*  = 60), non-MEN benign disease (including multinodular goitre, Graves’ disease and primary hyperparathyroidism) (*n*  = 120) and those where the pathology was unclear (*n*  = 7). These groups were further classified as PoSH and non-PoSH (Table 1), and participants where the pathology was unknown were excluded from further analyses.

**Table 1:** Number of responses to the survey stratified by pathology, gland(s) operated and PoSH vs non-PoSH

Gland(s) operated	Groups			
	Thyroid cancer	MEN	Non-MEN and benign	Unsure
Thyroid				
PoSH	31	5	17	0
Non-PoSH	192	6	64	0
Parathyroid				
PoSH	0	11	1	0
Non-PoSH	0	20	31	0
Both				
PoSH	10	11	2	0
Non-PoSH	19	7	5	0
Unsure				
PoSH	0	0	0	1
Non-PoSH	0	0	0	6

Responders were predominantly female (female *n* = 379, male *n* = 52, undisclosed *n* = 8) with a median (range) age of 52 (19–92) years. The reported dates of surgery where there was potential for PoSH as a complication ranged from 1973 to 2019.

Of 439 responses, 347 had a fully complete SF-36 score.

**Fig. 2:**
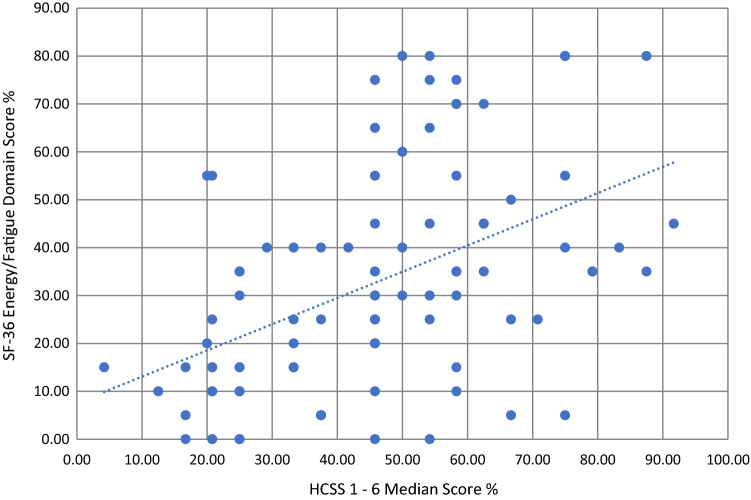
Graph depicting moderate correlation between hypocalcaemia symptom score (HcSS) and energy/fatigue domain of the SF-36 in 88 patients with PoSH (Spearman’s rho correlation coefficient 0.497, *p* < 0.001). Scores range from 100 (asymptomatic/good health) to 0 (symptomatic/poor health)

The median (interquartile range) reported QoL in the overall dataset (*n* = 439) between PoSH and non-PoSH groups was 54.2% (37.2—76.8%) and 60.6% (39.9—79.1%), respectively (Mann–Whitney U test; *p* = 0.348). Overall and domain-specific SF-36 scores in the various groups stratified by pathology and presence of PoSH are shown in Fig. 2. Across all pathologies, there was no significant difference in scores between PoSH and non-PoSH in any of the eight domains except the energy/fatigue (VT) subdomain where the scores were significantly lower in the PoSH group (*p* = 0.008). When stratified by pathology (Table 2), VT was the only domain showing a significant difference in the thyroid cancer (*p* = 0.032) and MEN group (*p* = 0.029), but not in the benign group (*p* = 0.620). The overall SF-36 score and the domain scores in patients across the different pathological groups (Thyroid cancer, MEN and benign) are compared in Fig. 2.

**Table 2:** Comparison of the overall SF-36 score and its domains in patients across groups stratified by pathology

	Thyroid cancer *n* = 252	MEN syndrome *n* = 60	Benign pathology *n* = 120	Significance (Kruskal–Wallis’ test)
SF-36	62.6 (44.4–79.9)	61.2 (33.8–83.5)	48.5 (31.5–68.9)	<0.001
PF	85 (60–100)	90 (44.3–100)	65 (31.3–90)	<0.001
RP	50 (0–100)	25 (0–100)	25 (0–100)	0.023
RE	66.7 (0–100)	83.3 (0–100)	33.3 (0–100)	0.154
VT	40 (25–60)	40 (18.8–60)	30 (10–50)	0.003
MH	65 (48–80)	66 (44–81)	60 (40–72)	0.010
SF	75(50–100)	62.5 (50–88)	62.5 (38–75)	<0.001
BP	67.5 (45–100)	67.5 (35–90)	55 (34.5–78)	<0.001
GH	50 (25–70)	40 (20–65)	35 (20–53.8)	<0.001

Median (25th and 75th percentile) of percentage SF-36 score. SF-36 = overall SF-36 score, PF = physical functioning, RP = role limitations due to physical health, RE = role limitations due to emotional problems, VT = energy/fatigue (vitality), MH = emotional well-being (mental health), SF = social functioning, BP = bodily pain, GH = general health

**Fig. 3:**
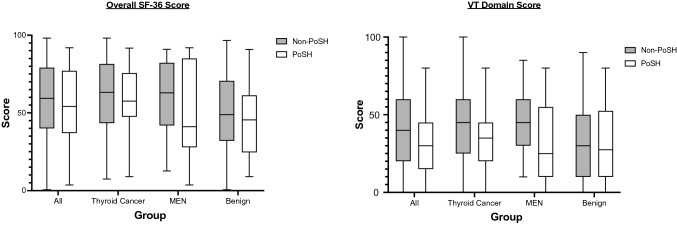
Comparison of the overall SF-36 score and the VT (energy/fatigue) domain in patients with and without PoSH in the overall cohort and groups stratified by pathology. Scores range from 100 (asymptomatic/good health) to 0 (symptomatic/poor health)

Of the respondents to the HcSS (*n* = 419), participants with PoSH (*n* = 88) reported significantly more symptoms associated with hypoparathyroidism than those without (*n* = 331). As shown in Fig. 3, HcSS were significantly lower in participants with PoSH compared to those without in the overall comparisons (*p* < 0.001) and in subgroups of thyroid cancer (*p* = 0.004), MEN (*p* = 0.006) and benign (*p* = 0.019) cohorts.

The median SF-36 and HcSS outcomes were compared by decade in patients where the year of operation was provided (Table 3). There was no statistically significant difference in either the SF-36 or HcSS scores across the decades for patients with PoSH. The SF-36 and HcSS scores varied within the non-PoSH group with patients having had operations in the years 2000 to 2009 showing the highest outcomes for quality of life and least symptoms of hypoparathyroidism and those having had operations in the years 2010 to 2019 reporting the lowest quality of life and highest symptoms in the HcSS. These differences were found to be statistically significant using the Kruskal–Wallis test (SF-36 *p* = 0.005, HcSS *p* = 0.002).

**Table 3:** Comparison of overall SF-36 and HcSS scores in patients with and without PoSH across decades

	Decade			Significance (Kruskal–Wallis’ test)
	> 1999	2000–09	2010–19	
SF-36				
PoSH	59.2 (29.2–84.3) *n* = *10*	50.8 (39.0–75.1) *n* = *27*	56.2 (35.5–75.9) *n* = *50*	0.935
Non-PoSH	68.4 (41.4–86.1) *n* = *12*	73 (47.6–84.7) *n* = *81*	56.3 (37.4–75.6) *n* = *244*	0.005
HcSS				
PoSH	45.8 (27.1–67.7) *n* = *10*	45.8 (25.0–58.3) *n* = *26*	45.8 (30.2–58.3) *n* = *50*	0.854
Non-PoSH	62.5 (58.3–83.3) *n* = *11*	66.7 (45.8–75.0) *n* = *78*	54.2 (41.7–68.2) *n* = *299*	0.002

Figure legend: median (25th and 75th percentile) of percentage SF-36 and HcSS score

Of the 89 responders with PoSH, 88 had their calcium monitored within the last 6 months. Only 43% reported that their calcium was within the normal range. 44% had ‘low’ or ‘very low’ calcium and 3% reported a ‘high’ calcium. Most patients (92%) with PoSH also had a PTH tested, though fewer patients were able to recall the result. 39% recalled their PTH to be ‘low’ or ‘very low’ and no patients had a ‘high’ PTH. Data from the STHNFT patients were validated. Eighty STHNFT patients had provided consent and enough identifiable information to review their medications and blood test results. All 11 patients who self-identified to have PoSH were prescribed alfacalcidol. Of 7 patients with PoSH who reported their calcium to be ‘low’ or ‘normal’, five patients were correct. One patient reported ‘normal’ calcium and had a low calcium and PTH, one reported ‘low’ calcium but had a low PTH with normal calcium. Eight patients had stated they were on treatment for hypocalcaemia but were not on active vitamin D on the medication list they provided. One of these patients was prescribed alfacalcidol by the GP. All other patients were on low dose vitamin D supplements or calcium for bone health.

## Discussion

This study is the largest match control study to date of QoL in patients with PoSH. Patients with PoSH are reported to have an impaired QoL in several recent studies [12–16, 22] and a systematic review [11]. The majority of the studies (Table 4) have used validated QoL questionnaires such as the SF-36 and found that patients with PoSH have significantly reduced QoL compared to the general population or normative datasets. This comparison may not be valid as PoSH occurs in the context of underlying benign or malignant thyroid and/or parathyroid disease, which will have an impact on QoL.

**Table 4:** Summary of recent Quality of Life (QoL) studies on patients with post-surgical hypoparathyroidism (PoSH)

Reference	Population	Definition of PoSH	Control	QoL measures	Key findings	Limitations
Arlt, Fremerey et al. 2002 [15]	Women with PoSH after goitre surgery or parathyroidectomy for PHPT (*n* = 25); PoSH on stable treatment	Treatment with calcium and active vitamin D supplements for > 6 months following surgery	Women without PoSH after thyroid surgery (*n* = 25); matched for age and time since surgery	ACL-90-R; GBB-24; and B-L Zerssen	Higher global complaint score (GBB-24 *p* = 0.036, B-L Zerssen *p* = 0.002, SCL-90-R *p* = 0.020) Predominant increase in anxiety	Small study numbers, parathyroid disease excluded
Astor, Løvås et al. 2016 [12]	PoSH documented by ICD-10 codes (*n* = 197, *F* = 161)—preceding surgery undefined	Treatment (undefined) for > 1 year following surgery where serum calcium below reference range with simultaneously low or inappropriately normal PTH	Norwegian national normative data	SF-36 HADS	Significantly lower SF-36 scores and higher symptom score for anxiety and depression in PoSH patients	Unmatched controls Surgical intervention is not defined
Sikjaer, Rolighed et al. 2014 [13]	Total (*n* = 62 F = 53), PoSH (n = 58)—atoxic goitre (* n* = 25), toxic goitre (* n* = 17) Cancer (*n* = 12), PHPT (*n* = 4)	Treatment with active vitamin D or high dose D2 for > 1 year where serum calcium below reference range with simultaneously low or inappropriately normal PTH	US National normative data	SF-36 WHO-5	PoSH patients had significantly lower scores in all domains (*p* < 0.05). No significant difference in WHO-5 scores	Surgical intervention not defined. No matched controls
Sikjaer, Moser et al. 2016 [16]	PoSH post-thyroidectomy (*n* = 22, *F* = 3)	Treatment with active vitamin D or high dose D2 for > 1 year where serum calcium below reference range with simultaneously low or inappropriately normal PTH	Post-thyroidectomy without PoSH (*n* = 22), healthy Individuals matched for sex and age at time of testing (± 2 years) and time of surgery (± 2 years) (*n* = 22)	SF-36 WHO-5	PoSH had lower scores in all SF-36 domains except role emotional subdomain. WHO-5 showed no difference between the two surgical groups (*p* = 0.72), but both had a lower score than the controls (*p* < 0.01)	Small sample size
Cusano, Rubin et al. 2013 [14]	Total (n = 54, F = 40), PoSH (n = 27) Surgical intervention undefined	Treatment with calcium and vitamin D for > 6 months for serum calcium and PTH concentrations below the lower limits of normal on at least 2 occasions separated by at least 30 days Chronic HPT defined as > 2 years	US National Normative data	SF-36	Significantly lower scores in all subdomains (*P* < 0.001)	Small sample size, no matched controls Surgical intervention undefined
Wilde, Wilken et al. 2020 [22]	PoSH following thyroid surgery (*n* = 49, *F* = 36) (Total thyroidectomy/subtotal/near total/hemi and subtotal)	Presence of hypocalcaemia and inappropriately low PTH levels and patients needing treatment (undefined) at least 6 months after surgery	39 following thyroid surgery (as for population and hemithyroidectomy) 35 hyperparathyroidism matched for sex and age	HPQ 40/28	Identification of specific symptoms in patients with HPT differed to controls	Unvalidated tool Extent of surgery not matched, i.e. total thyroidectomy and hemithyroidectomy. Underlying pathology not matched, e.g. carcinoma, Graves’ disease

Matched control studies only include very small numbers of patients: Arlt et al. compared 25 females with PoSH with 25 females without PoSH [15], while Sikjaer et al. compared 22 PoSH patients post-thyroidectomy with 22 post-thyroidectomy and 22 healthy individuals without PoSH [16].

Studies comparing SF-36 in patients with PoSH to normative data show that patients with PoSH report a significantly decreased QoL in at least 7 out of 8 domains [12–14]. In the study by Sikjaer et al. where appropriate control groups were used, there are fewer differences in reported QoL [15, 16]. In this study, when patients with PoSH are compared to normative data, QoL is significantly lower in 7 out of 8 domains of the SF-36. In contrast, when compared to matched controls there is only a significant (*p* < 0.05) difference in 2 out of 8 domains [16].

Studies have considered four main factors which impair QoL: being in a follow-up programme, the effect of the disease on daily activities, coming to terms with the diagnosis and uncertainty concerning the future [23].

This study evaluated 439 patients who have had surgical treatment that could predispose them to PoSH and compared QoL in those with and without PoSH. QoL may be influenced by factors including underlying pathology, coexisting morbidity, ongoing management and other treatment related morbidity. For this reason, cohorts chosen from different sources were categorised into groups primarily on the basis of underlying pathology—thyroid cancer, MEN syndromes and others.

**Fig. 4:**
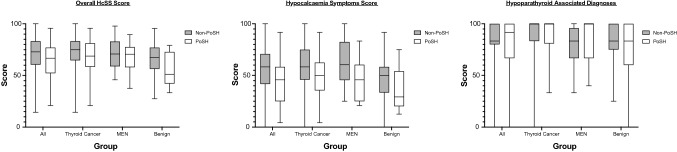
Comparison of the overall HcSS score and its components in patients with and without PoSH in the overall cohort and groups stratified by pathology. Scores range from 100 (asymptomatic/good health) to 0 (symptomatic/poor health)

The results show that hypocalcaemia-related symptoms are worse in patients with PoSH compared to those without. We have also shown that patients with PoSH report a significantly lower level of energy and increased fatigue. As shown in Fig. 4, there was moderate correlation between the HcSS questions regarding symptoms of hypocalcaemia and the energy/fatigue (VT) domain of the SF-36 (Spearman’s rho correlation coefficient = 0.497, *p* < 0.001). This suggests that issues related to calcium balance is associated with low energy levels.

Patients with PoSH reported a higher rate of renal calculi (*p* = 0.041) or renal failure (*p* < 0.001): conditions closely associated with long-term hypoparathyroidism [2]. Other diagnoses (such as seizures, renal failure and cataracts) have been described as occurring more frequently in patients with PoSH, compared to the general population [2, 24, 25]. In this study, the reported incidence of these other conditions did not differ between the PoSH and non-PoSH groups, another indicator that comparing groups that are otherwise similar (bar the diagnosis of PoSH) reveals the true impact of PoSH.

Treatment of patients with PoSH is primarily aimed to address symptoms. The cause for persistence of symptoms could be due to either inadequate or ineffective treatment, low compliance or a combination of these issues. Guidelines on the management of PoSH emphasise the importance of keeping the calcium in the low normal range [26] and this may partly account for ongoing symptoms in some patients. It is possible that increasing the dose of calcium and/or vitamin D may improve symptoms in these individuals, but this may increase risk of hypercalcaemia, hypercalciuria and its associated long-term morbidity.

Despite patients with PoSH having significantly more symptoms compared with the non-PoSH group and a moderate correlation between the degree of symptoms on HcSS and energy/fatigue scores (Fig. 4), this has not translated into a significant reduction in overall QoL. This may either be due to small sample size or due to the impact of other comorbidity in these patients. However, there was a consistent and statistically significant reduction in scores in the energy and fatigue domain as reported by patients with PoSH.

The surgeon’s awareness of the importance of parathyroid preservation during thyroid surgery has significantly increased over time [27]. This is reflected in surgical techniques, use of novel technologies and improved patient monitoring. Practices such as routine parathyroid auto-transplantation, actively ‘seeking’ the parathyroid glands and routine subtotal thyroidectomy have been unsuccessful in reducing PoSH [28, 29]. The current recommendation is that glands should be identified and preserved, but if their vascular supply is compromised then auto-transplantation into a muscle is recommended during thyroidectomy [30]. In patients with parathyroid disease, pre-operative localisation has improved, and patients are undergoing less invasive, unilateral or targeted operations, reducing the risk of PoSH.

Several novel technologies have been developed over the past 10 years to augment parathyroid identification. Technologies such as ICG fluorescence aim to aid identification and prevent damage to the glands with the potential to improve patient outcomes and QoL. The use of routine post-operative biochemical testing for early detection, management and monitoring of patients with PoSH both in the short term [31] and in the long term [26] also indicates an improved understanding of the incidence and potential morbidity of this condition. Awareness of vitamin D deficiency, pre-operative correction and careful post-operative monitoring enables timely initiation of treatment and appropriate follow-up. This can treat patients before the symptoms of hypocalcaemia become severe. Most patients will be able to cease treatment soon after or at least within six months of surgery [7, 32]. Careful follow-up of patients with monitoring of calcium, magnesium, phosphate, vitamin D, PTH and renal function with ongoing adjustments to their medications is important to ensuring adequate control of symptoms and limit morbidity of over treatment [33].

This is a questionnaire study subject to selection and recall bias as with other similar studies. Clinical details provided by participants were not corroborated, potentially introducing errors in interpretation. Allocation to PoSH or non-PoSH groups was based on participants’ recall of their medications.

This large, matched case–control study of QoL in patients with PoSH has shown a significant impact on patients’ energy levels and fatigue. Overall, patients with PoSH had more symptoms of paraesthesia, muscle cramps and diarrhoea. No significant difference was found in overall QoL in the SF-36 between the PoSH and non-PoSH groups. The SF-36 VT domain score showed moderate correlation with the symptoms of hypocalcaemia. A disease-specific tool may identify further aspects of quality of life not explored as part of the SF-36. We are not in the process of validating the HcSS, but these initial results do highlight that there are symptoms patients suffer with that are not included in the SF-36.

Given the potential for persistence of symptoms despite treatment, deterioration in energy levels and long-term morbidity, further research to reduce the occurrence of PoSH through improvements in surgical techniques and use of novel technologies should be facilitated.
